# Novel approaches to the prediction, diagnosis and treatment of cardiac late effects in survivors of childhood cancer: a multi-centre observational study

**DOI:** 10.1186/s12885-017-3505-0

**Published:** 2017-08-03

**Authors:** Amy Skitch, Seema Mital, Luc Mertens, Peter Liu, Paul Kantor, Lars Grosse-Wortmann, Cedric Manlhiot, Mark Greenberg, Paul C Nathan

**Affiliations:** 1 St. Michael’s Hospital, 30 Bond Street, Toronto, ON M5B 1W8 Canada; 2 The Hospital for Sick Children, 555 University Avenue, Toronto, ON M5G 1X8 Canada; 3 University of Toronto, Toronto, Canada; 4 University of Ottawa Heart Institute, 40 Ruskin Street, Ottawa, ON K1Y 4W7 Canada; 5 Stollery Children’s Hospital, 8440 112 Street Northwest, Edmonton, AB T6G 2B7 Canada; 6 University of Alberta, Edmonton, Canada; 7 Pediatric Oncology Group of Ontario, Toronto, Canada

**Keywords:** Childhood cancer, Cardiac, Late effects, Treatment, Survival, Anthracycline therapy

## Abstract

**Background:**

Anthracycline-induced cardiac toxicity is a cause of significant morbidity and early mortality in survivors of childhood cancer. Current strategies for predicting which children are at greatest risk for toxicity are imperfect and diagnosis of cardiac injury is usually made relatively late in the natural history of the disease. This study aims to identify genomic, biomarker and imaging parameters that can be used as predictors of risk or aid in the early diagnosis of cardiotoxicity.

**Methods:**

This is a prospective longitudinal cohort study that recruited two cohorts of pediatric cancer patients at six participating centres: (1) an Acute Cohort of children newly diagnosed with cancer prior to starting anthracycline therapy (*n* = 307); and (2) a Survivor Cohort of long-term survivors of childhood cancer with past exposure to anthracycline (*n* = 818). The study team consists of three collaborative cores. The *Genomics Core* is identifying genomic variations in anthracycline metabolism and in myocardial response to injury that predispose children to treatment-related cardiac toxicity. The *Biomarker Core* is identifying existing and novel biomarkers that allow for early diagnosis and prognosis of anthracycline-induced cardiac toxicity. The *﻿Imaging Core* is identifying echocardiographic and cardiac magnetic resonance (CMR) imaging parameters that correspond to early signs of cardiac dysfunction and remodeling and precede global dysfunction and clinical symptoms. The data generated by the cores will be combined to create an integrated *risk-prediction model* aimed at more accurate identification of children who are most susceptible to anthracycline toxicity.

**Discussion:**

We aim to identify genomic risk factors that predict risk for anthracycline cardiotoxicity pre-exposure and imaging and biomarkers that facilitate early diagnosis of cardiac injury. This will facilitate a personalized approach to identifying at-risk children with cancer who may benefit from cardio- protective strategies during therapy, and closer surveillance and earlier initiation of medications to preserve heart function after cancer therapy.

**Trial registration:**

NCT01805778. Registered 28 February 2013; retrospectively registered.

**Electronic supplementary material:**

The online version of this article (doi:10.1186/s12885-017-3505-0) contains supplementary material, which is available to authorized users.

## Background

With contemporary therapies, over 80% of children diagnosed with cancer will become long-term survivors [1, 2]. The childhood cancer survivor (CCS) population in the United States exceeds 390,000 [3]. CCS are at significant risk of serious morbidity and premature mortality as a result of their cancer therapy [4, 5]. Cardiac toxicity, mainly caused by anthracycline chemotherapy agents (e.g. doxorubicin, daunomycin) which are administered to more than 50% of children with cancer [6], is a major cause of this morbidity. Although observed frequencies vary between studies, up to 60% of patients treated with an anthracycline will develop echocardiographic abnormalities [7]. These abnormalities increase over time in incidence and severity in a significant proportion of patients [8–10]. The risk of congestive heart failure (CHF) in children exposed to a cumulative anthracycline dose greater than 300 mg/m^2^ approaches 10% by 20 years after their cancer therapy [11], but even children exposed to lower doses of anthracyclines are at significantly increased risk for CHF [7, 12]. Compared to their siblings, CCSs have a 15-fold increased risk of developing CHF [13]. Cardiac disease is the third leading cause of premature death in CCS (after cancer recurrence and second malignancies), with a 7-fold increased risk of premature cardiac death as compared to the general population. The relative risk of cardiac death remains elevated even in CCS who have survived for more than 25 years after their primary cancer [14].

Clinicians rely on established clinical risk factors (e.g. cumulative anthracycline dose, radiation therapy to a field that involves the heart, younger age at treatment, female gender, longer follow-up, and CHF during therapy [7–9, 15–19]) in order to identify which children treated for cancer are at risk for late-onset cardiac dysfunction. The Children’s Oncology Group guidelines for surveillance for late effects in CCS recommend a surveillance echocardiogram or MUltiGated Acquisition scan (MUGA) every 1, 2, or 5 years depending on three risk factors: (1) age at treatment; (2) cumulative anthracycline dose; and (3) receipt of chest radiation [20–22]. However, these factors are imperfect predictors, and do not take into account individual biological variations in metabolism of chemotherapy and response to cardiac injury. Consequently, their discriminative power for individual patient decision making is poor [23].

Despite the identification of some genetic variants that predispose to anthracycline cardiotoxicity, genetic factors need further study and validation before they can be applied in clinical practice [24]. Different cardiac biomarkers could be used for the detection of subtle cardiac damage prior to the onset of imaging or functional changes, and may identify an “at risk” population that could benefit from modification of future chemotherapy, administration of a cardioprotectant or intervention aimed at the prevention of cardiac remodeling and progressive dysfunction [25]. Further validation of the utility of biomarker testing to predict individual risk of cardiac toxicity is required before it can be recommended for routine use.

Most commonly, cardiotoxicity is monitored using echocardiographic measures of systolic function including left ventricular (LV) ejection fraction (EF) or shortening fraction (SF). These parameters are frequently normal early in the natural history of anthracycline cardiomyopathy, even when biopsy studies demonstrate significant evidence of myocardial damage such as apoptosis and interstitial fibrosis [26]. More recently, novel echocardiographic parameters such as tissue Doppler echocardiography and strain imaging, have been shown in adults to detect early changes in cardiac function prior to changes in ejection fraction. There are still limited data in children on the utility of the new echocardiographic methods [27]. Other novel imaging techniques include the use of Cardiac Magnetic Resonance (CMR). Late gadolinium enhancement (LGE) is commonly used as an imaging biomarker of discrete myocardial scarring in cardiomyopathies [28], although its significance in anthracycline-induced cardiotoxicity is uncertain [29]. Using T1relaxometry based approaches, commonly referred to as ‘T1 mapping’, it is possible to measure myocardial extracellular volume (ECV), which has been shown to correlate with the degree of cardiac fibrosis [30, 31]. ECV has been found to be correlated with cumulative anthracycline dose, exercise capacity and myocardial wall thinning in a group of 30 adolescent patients at least 2 years following anthracycline treatment [32]. If these findings are confirmed and if ECV can be demonstrated to carry prognostic significance, it could serve as an early tissue marker of fibrotic ventricular remodeling, especially if it precedes decreased ejection fraction in children post-anthracycline therapy. Given the limitations of using SF/EF for the early detection of cardiac damage and the observation that CHF may not occur for years (or even decades) after anthracycline exposure, it is not feasible to use SF/EF or clinical cardiac disease as the sole outcome in studies of CCS during the pediatric years. There is a pressing need to develop more sensitive imaging and biomarker techniques that will allow for earlier detection of sub-clinical treatment-induced cardiac toxicity, and that can be combined with genetic predictors to identify survivors at greatest risk for progressive cardiac deterioration [33].

Here we report on the design and methods of the ‘*Novel approaches to the prediction, diagnosis and treatment of cardiac late effects in survivors of childhood cancer*’ study. To our knowledge, this is the first longitudinal pediatric cohort study to evaluate a combination of predictive variables in order to develop a risk prediction algorithm specific to CCS at risk for cardiac disease. We aim to:

1. Identify genetic predictors of anthracycline cardiotoxicity;
2. Assess existing biomarkers and identify novel biomarkers for the assessment of acute and chronic cardiac toxicity in children treated with anthracyclines;
3. Identify the echocardiographic and CMR parameters that best identify early cardiac changes and predict progressive cardiac deterioration after exposure to anthracyclines;
4. Create a statistical model that combines genomic, biomarker, imaging and clinical data to predict which pediatric patients exposed to anthracycline chemotherapy will develop progressive cardiac damage.

## Methods

This is a multi-centre observational cohort study that is being conducted at the Hospital for Sick Children (Toronto, Canada), Princess Margaret Cancer Centre (Toronto, Canada), McMaster Children’s Hospital (Hamilton, Canada), London Health Sciences Centre (London, Canada), The Children’s Hospital of Eastern Ontario (Ottawa, Canada) and The Children’s Hospital of Orange County (Orange County, USA). Ethics approval was obtained by the following Ethics Boards for the conduct of this study: The Hospital for Sick Children Research Ethics Board, University Health Network Research Ethics Board, University of Western Ontario Health Sciences Research Ethics Board, Children’s Hospital of Eastern Ontario Research Ethics Board, McMaster Health Sciences Research Ethics Board, and Children’s Hospital of Orange County In-House Research Ethics Board. Written informed consent was obtained from all study participants (or parent/legal guardian consent along with patient assent, where applicable).

Two patient cohorts were recruited and are being followed longitudinally at the six participating centres.

### Acute cohort

**Fig. 1:**
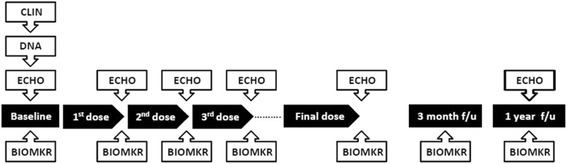
Data and specimen acquisition from the Acute Cohort. BIOMKR: Serum for biomarkers, ECHO: Echocardiogram, DNA: Blood or saliva for DNA, CLIN: Gather baseline clinical data

A prospective cohort of patients newly diagnosed with cancer who received anthracycline chemotherapy has been recruited from clinics at the four pediatric participating centres (recruitment target *n* = 270; recruitment actual *n* = 307). We will assess whether genetic predictors of anthracycline susceptibility, biomarkers of early cardiac damage, and imaging parameters of acute cardiac dysfunction predict which patients will demonstrate evidence of persistent or progressive cardiac damage at the 12 months follow-up from their last cycle of anthracycline chemotherapy (See Fig. 1 for timeline of sample and data acquisition in Acute Cohort). Eligibility criteria for both cohorts are provided in Table 1.

**Table 1:** Inclusion and exclusion criteria by cohort

	Inclusion criteria	Exclusion criteria
Acute Cohort	1. Aged <18 years at time of cancer diagnosis; 2. Diagnosed with a new malignancy; 3. Cancer treatment plan will require therapy with ≥1 dose of an anthracycline chemotherapy 4. Pre-anthracycline; echocardiograms to occur at the recruiting site; 5. Normal cardiac function prior to the initiation of anthracycline therapy (LVEF >55%).	1. Patients with significant congenital heart defects; 2. Patients who were previously treated with anthracycline chemotherapy or radiation to the chest.
Survivor Cohort	1. Aged <18 years at time of cancer diagnosis; 2. Previously diagnosed with cancer and currently in remission; 3. Patients whose prior treatment plan included therapy with ≥1 dose of anthracycline chemotherapy; 4. Patients who completed their final dose of anthracycline ≥3 years ago; 5. Patients who completed their final dose of a chemotherapy agent other than anthracycline ≥1 year ago; 6. Routinely followed at the recruiting site approximately every 12 months.	1. Prior allogeneic stem cell transplant; 2. Patients with significant congenital heart defects; 3. CMR: general contraindications for a contrast enhanced CMR, and patients who require anaesthesia for MRI (typically <6 years of age) will be excluded.

Family and medical history and demographics are collected at baseline. Data on concomitant medications are collected at each study visit. A blood (4-6 ml) or saliva (2 ml) sample is collected for DNA extraction and genomic analysis. Serial 2D echocardiograms are obtained at baseline, and at 12 months post-final anthracycline therapy dose. Additional echocardiograms are obtained prior to each dose of anthracycline in consenting patients. A blood sample (5-8 ml per time point) is collected prior to each dose of anthracycline therapy, and at 3 months and 12 months post the last anthracycline dose. The consent for biomarker studies is optional. In a subset of patients over 6 years of age, patients are also approached for consent for a cardiac MRI at the 12 month follow-up time point (Table 2).

**Table 2:** Schedule of procedures/evaluations in Acute Cohort

Procedure/Evaluation	Baseline (prior to starting anthracycline)	Study Visits prior to each anthracycline dose	3 months after completion of final anthracycline dose (± 4 weeks)	12 months after completion of final anthracycline dose (± 8 weeks)
Informed Consent/Assent	X			
Demographics	X			
Height (cm) & Weight (kg)	X	X	X	X
Concomitant Medications	X	X	X	X
Medical History	X			
Review of Medical Events	X	X	X	X
Cancer Therapy		X	X	
Echocardiogram (ECHO)	X	X^a^		X
Genetic Sample	Obtain one sample at any time point while patient is on-study^%^			
OPTIONAL Biomarker Sample	X	X	X	X

^%^For patients who will be having an allogeneic stem cell transplant, collect the genetic sample prior to the procedure

^a^ECHOs will be performed prior to each dose of anthracycline when possible. If patient/parent is not agreeable to research-only ECHOs, then they will occur only at clinically indicated time points

### Survivor cohort

**Fig. 2:**
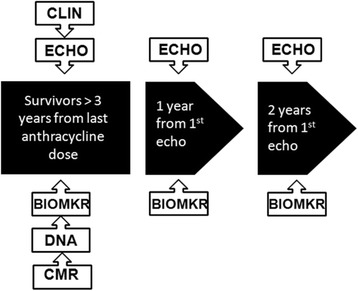
Data and specimen acquisition from the Survivor Cohort. BIOMKR: Serum for biomarkers, ECHO: Echocardiogram, DNA: Blood for DNA, CMR: Cardiac Magnetic Resonance, CLIN: Gather baseline clinical data

The survivor cohort consists of childhood cancer survivors who are three or more years from their last cycle of anthracycline therapy (recruitment target *n* = 920; actual recruitment *n* = 818). This cohort has been recruited from specialized survivor clinics at the six participating centres. A blood or saliva sample for DNA is collected at enrollment. Echocardiogram to assess function and blood sample for biomarkers are obtained at enrollment and annually over 2 years of follow-up (see Fig. 2 for timeline of sample and data acquisition in Survivor Cohort).

Family and medical history, cancer therapy history and demographics are collected at baseline. Concomitant medications are collected at each study visit. All consented patients agree to provide a sample for genomics as well as serial echocardiograms at enrollment, and 12 and 24 months from baseline. Biomarker consent and consent for CMR are optional. Participants who consent to biomarkers provide 5-8 mL of blood at the time of each echocardiogram. Participants who consent to the CMR component of the study have a CMR at any one of the enrollment, 12 month or 24 month study time points (Table 3).

**Table 3:** Schedule of Procedures/Evaluations in Survivor Cohort

Procedure/ Evaluation	Baseline (0 months)	Month 12 (± 8 weeks)	Month 24 (± 8 weeks)
Informed Consent/ Assent	X		
Demographics	X		
Cancer Therapy History	X		
Height (cm) & Weight (kg)	X	X	X
Medication and medical event review	X	X	X
Echocardiogram (ECHO)	X	X	X
Genetic Sample	Obtain one sample at any time point while patient is on-study		
OPTIONAL Biomarker Sample	X	X	X
OPTIONAL CMR	Occurs at one time only while patient is on-study.^a^		

^a^CMR will be performed in a subset of consented study patients only

### Data management

Demographic, treatment and outcome data is captured at each site and entered directly into a secure web-based application known as REDCap (Research Electronic Data Capture [34]). Patients have been assigned a unique subject number upon enrolment into the study. This is assigned at the site and registered in REDCap. Data entered into the REDCap database is de-identified at each recruiting centre through use of a unique study identifier. Study data entered into REDCap is verified by the coordinating centre at the Hospital for Sick Children and any inconsistencies or queries rre sent to the appropriate site coordinator for resolution in the REDCap database. REDCap maintains a built-in data verification feature as well as a built-in audit trail that logs all user activity and all pages viewed by every user (https://redcapexternal.research.sickkids.ca/). This will allow the coordinating centre to determine all the data entered, viewed or modified by any given user.

### Study outcomes

Based on paediatric and adult data that show that early cardiac remodelling precedes global dysfunction, the study will use remodeling parameters (left ventricular posterior wall thickness (LVPWT) Z-score and LV thickness to dimension ratio (TDR)) as markers of early cardiac injury that can identify patients who are at risk for progressive cardiac dysfunction later in life. Thus, the primary outcome measurement in each of the genomics, biomarker and imaging cores is the presence of one or more of the following at 12 months after anthracycline in the Acute Cohort, or at any study time point in the Survivor Cohort:

- Cardiac remodelling defined as an LVPWT or TDR z-score < −2.0 (or a reduction in LVPWT or TDR z-score by ≥1 standard deviation compared to baseline in the Acute group); or
- Reduced LV EF (<55%) or a drop in LV EF of ≥10% over serial echocardiograms; or
- Symptomatic heart failure graded using New York Heart Association (NYHA) classification (or Ross heart failure class 2 in infants <2 years old).

This study is being conducted by three collaborative cores:

### CORE 1: Genomics

The Genomics Core will perform a comprehensive genome-wide search to identify genes associated with anthracycline cardiotoxicity.

The study will use a nested case-control approach. Patients who are anthracycline sensitive (i.e. develop cardiac dysfunction despite low anthracycline doses), and those who are anthracycline resistant (i.e. have preserved cardiac function despite high anthracycline doses) will be included in a discovery cohort. These patients will undergo whole exome sequencing to identify genes associated with cardiotoxicity. Genes in pathways related to anthracycline absorption, distribution, metabolism, and excretion, and genes important in cardiac response to injury will be prioritized in the analysis. Non-genetic risk predictors will be included in the regression model. The top-ranked genes that are enriched for variants and are deemed biologically relevant will undergo targeted sequencing in the remainder of the cohort which will serve as a replication cohort.

### CORE 2: Biomarkers

The Biomarker Core will explore whether existing and novel biomarkers allow for more accurate diagnosis of acute and late treatment-related cardiac toxicity.

#### Aim 1 (acute cohort)

To determine and validate which of the currently available dynamic protein biomarkers detectable in serum during the acute phase of anthracycline administration can predict early cardiac remodeling.

A panel of biomarkers that have been shown to indicate cardiac stress or injury, including N-terminal pro b-type natriuretic peptide (NTproBNP), high sensitivity troponin (hsTnT), myeloperoxidase (MPO), and insulin-like growth factor binding protein 7 (IGF-BP7) will be evaluated. The discovery cohort will be derived from the children enrolled in the Acute Cohort at The Hospital for Sick Children. The validation cohort will be derived from similarly recruited patients in the Acute Cohort from London, Ottawa and Hamilton. Patients will have serum collected at the time points specified in Fig. 1. The serial serum samples will be assayed for levels of the target markers at each of the collection time points. Both the individual marker levels and the patterns of change over time will be evaluated against the primary outcome (evidence of remodeling, decreased EF or CHF at 1 year). The candidate biomarkers will be evaluated using quality controlled assays on the most appropriate platforms (e.g. for NTproBNP, hsTnT and IGF-BP7, the Roche Elecsys platform available in the Biomarker Core laboratory will be used, and for MPO, standard human serum ELISA kits such as that provided by Eagle Bioscience, NH will be used). These will be performed in replicate with appropriate controls. The best candidates from the discovery cohort will then be applied to the validation cohort to determine the reproducibility and cross population validity of the marker performance.

#### Aim 2 (survivor cohort)

To determine the correlation of currently available biomarkers that can be detected in the serum of cancer survivors with imaging parameters of cardiac remodeling or dysfunction.

In order to determine which biomarkers correlate with remodeling and sub-clinical dysfunction in survivors (>3 years from anthracycline chemotherapy), the same panel of biomarkers being assessed in the acute cohort samples will be evaluated. Serum will be collected from the Survivor Cohort concurrent with study echocardiograms at baseline, 1 year and 2 years (Fig. 2). Cases will be defined as patients in the Survivor Cohort demonstrating evidence of cardiac remodeling, EF < 55% (or a drop in EF of ≥10%) or CHF at any of the three study visits. The panel of biomarkers will be evaluated using the standardized methodology described above for the acute cohort.

### CORE 3: Cardiac imaging

The Cardiac Imaging Core will focus on the evaluation of new echocardiographic and CMR imaging techniques aimed at early identification of cardiac damage after anthracycline exposure. It will investigate whether changes in cardiac function immediately after anthracycline administration predict which patients will develop progressive cardiac dysfunction over time, and it will explore disease progression through the longitudinal evaluation of innovative echocardiographic parameters of remodeling and dysfunction in CCS exposed to anthracyclines. The Cardiac Imaging Core will also assess CMR markers of discrete as well as diffuse fibrotic myocardial remodeling late after anthracycline exposure and their relationship with diastolic and systolic ventricular function in a subset of eligible patients.

#### Aim 1 (acute cohort)

The core will determine whether reduced myocardial strain measurements are observed after acute anthracycline exposure and whether these changes in strain parameters predict the occurrence of adverse cardiac remodeling (as measured by changes in LV dimension and wall thickness) or dysfunction (as measured by change in ejection fraction from baseline to 12 months after therapy).

A baseline echocardiogram will be completed prior to commencing cancer therapy using a standardized functional protocol (Additional file 1). The protocol will be repeated prior to each anthracycline administration (which varies depending on each patient’s cancer treatment protocol), and at 12 months after the last dose of anthracycline chemotherapy, as outlined in Fig. 1. All images will be sent electronically to the imaging core laboratory (The Hospital for Sick Children) for centralized analysis. To standardize image acquisition at the different sites, training of the individual sonographers was provided by the core laboratory. Based on M-mode and 2-D echocardiography, LVEF by biplane Simpson’s, LVPWT and LV TDR z-score will be measured and calculated as recommended by the paediatric quantification guidelines issued by the American Society of Echocardiography [35]. Speckle tracking echocardiography is used to measure strain measurements as described by Koopman et al. [36]. Mean circumferential strain will be calculated from the basal short axis views. Circumferential strain measurements obtained in 6 segments will be averaged. Mean longitudinal strain measurements will be obtained from the apical 4-chamber view. Mean values in 6 segments will be averaged. Secondary imaging parameters including diastolic function parameters, tissue Doppler measurements and other myocardial deformation measurements will also be obtained. These will be analyzed to evaluate their usefulness for the early detection of myocardial dysfunction.

#### Aim 2 (survivor cohort)

The core will identify a sub-group of long-term survivors of childhood cancer with early signs of cardiac dysfunction, and describe the relationship of these parameters to cardiac remodeling parameters and biomarkers of cardiac damage. The trajectory of early dysfunction and remodeling over time will be examined in order to define a cardiac phenotype of early damage that can be a target of future intervention studies.

All patients enrolled in the Survivor Cohort will undergo an echocardiogram at baseline, 12 and 24 months (Fig. 2). At each of the three time points, LVPWT, TDR, and mean circumferential and longitudinal strain measurements will be performed. Subclinical dysfunction will be defined as a mean circumferential strain measurement at the basal level of the heart > − 15% or mean longitudinal strain > − 18%. The proportion of patients with evidence of sub-clinical dysfunction (assessed by strain), global dysfunction (EF < 55% or CHF) or remodeling (assessed by LVPWT, TDR) will be ascertained at each time point, and the rate of change in each parameter will be assessed over time The relationship between the remodeling parameters and strain parameters and the temporal relationship between changes in these parameters will be studied.

#### Aim 3 (acute and survivor cohorts)

The core will determine whether markers of diffuse or discrete myocardial fibrosis by T1 mapping CMR are associated with echocardiographic parameters of cardiac dysfunction and biomarkers of collagen metabolism.

Extracellular volume fraction (ECV) and native T1 time in the myocardium are both elevated in states of extracellular matrix expansion, as in diffuse myocardial fibrosis. In a pilot study,

Sixty patients from the survivor cohort will undergo one single CMR examination at any one of their three study visits. The CMR will include ECV and T1 measurements as well as assessment of ventricular volumes and ejection fraction. Discrete myocardial scarring will be assessed by means of LGE. ECV and T1 will be correlated with echocardiographic markers of systolic and diastolic myocardial and ventricular function. In addition, patients will be grouped into those with normal and those will abnormal diastolic function, as indicated by the mitral valve inflow and pulmonary vein profiles.

### Developing a risk prediction model

To achieve the primary objective of the Acute Cohort study (identifying patients at increased risk for therapy-induced cardiac disease prior to starting or during therapy), evolution of echocardiographic parameters over the treatment duration will be modeled in linear and non-linear regression models adjusted for repeated measures through a compound symmetry covariance structure. The resulting general estimating equations (GEE) will provide an estimate of the effect of anthracyclines over time for the entire cohort and the parameter estimate (slope of change over time) for each individual patient will be used as a potential predictor of therapy-induced cardiac damage. Twelve months after their final cycle of anthracycline chemotherapy, patients will be classified as having cardiac disease or not based on evidence of remodeling or decreased EF or CHF. Potential factors associated with acute cardiac change or cardiac damage at 12 months after treatment will be sought from patient demographics (e.g. age at treatment, gender), treatment (e.g. cumulative anthracycline dose, radiation exposure), genomics, serial biomarkers and echocardiographic measurements at baseline and during follow-up. Because of the very large number of potentially associated factors (and the multiple variations in format/time points for many factors), we will have to reduce the number of candidate factors using a pre-specified algorithm. As exploratory analyses, univariate regression models using therapy-induced cardiac changes or cardiac disease at 1 year (present vs. absent) as the dependent variable, and all other collected variables as potential independent variables will be created. This will allow exploration of variables potentially associated with therapy-induced cardiac damage including assessment of collinearity, ill condition (variables with no events in one of the study groups) or amount of missing data. All variables with univariable *p*-value <0.30, excluding collinear variables, ill conditioned variables or variables with an unacceptable amount of missing data will be included in a bootstrap bagging algorithm. A total of 5000 random sub-samples will be created and for each of those, a mixed stepwise regression model will be used to obtain multivariable predictors for therapy-induced cardiac damage. The proportion of sub-samples in which a given variable is selected is called reliability. Variables with high reliability (>50%) will then be included in a multivariable regression model with backward selection of variables to obtain a final model. This algorithm significantly improves the accuracy of variable selection, reduces the probability of sampling bias and corrects for multiple comparisons better than a post-hoc analysis would. At the end of this algorithm we will be left with a limited number of associated factors, all with high reliability. Based on the reliability estimates, we will be able to determine whether the risk of therapy-induced cardiac changes or cardiac damage at 1 year after anthracycline exposure is driven by baseline measurements (including clinical, genomic, echocardiographic or biomarkers), by reaching specific milestones during anthracycline treatment, by change over time in cardiac dimension/function and biomarkers during anthracycline treatment, or by a combination of all three.

## Discussion

Cardiac disease is the third leading cause of premature death in CCS, with a 7-fold increased risk of premature cardiac death as compared to the general population [14]. By the time clinical or imaging evidence of cardiac dysfunction becomes apparent, it is often late in the natural course of the disease making it difficult to intervene and reverse existing damage. The need to develop more sensitive techniques that will allow for earlier detection of anthracycline-induced cardiac toxicity is critical.

Findings from this research study may be able to inform clinical decision making through a risk prediction algorithm that will assist in identifying patients who are at an increased risk of anthracycline-induced cardiac toxicity. It is likely that combining genetic predictors of susceptibility with clinical risk factors will allow for a more personalized approach to identifying at-risk patients prior to initiating anthracycline therapy. This will allow for the modification of cancer therapy to prevent or reduce the risk of cardiac disease, allowing for optimal long-term outcomes in this patient population.

This cohort will provide an unparalleled resource for future research by providing not only a data resource, but also a risk prediction model, that will enable other investigators with an interest in cardiac late effects resulting from childhood cancer treatments to perform further investigation in the field.

## Additional file

Additional file 1: Echocardiographic Protocol. The standardized echocardiographic protocol outlines all images to be obtained in order to meet the study objectives in the Cardiac Imaging Core. (DOCX 13 kb)

## Abbreviations

CCS: Childhood cancer survivor
CHF: Congestive heart failure
CMR: Cardiac Magnetic Resonance
ECV: Myocardial extracellular volume
EF: Ejection fraction
GEE: General estimating equations
LV: Left ventricular
LVEF: Left ventricular ejection fraction
LVPWT: Left ventricular posterior wall thickness
REDCap: Research Electronic Data Capture
SF: Shortening fraction
SNPs: Single nucleotide polymorphisms
TDR: Thickness to dimension ratio
