# Supplementary material for: Novel approaches to the prediction, diagnosis and treatment of cardiac late effects in survivors of childhood cancer: a multi-centre observational study
Source: BMC Cancer. 2017 Aug 3;17:519. doi: 10.1186/s12885-017-3505-0 (PMC5543740; doi:10.1186/s12885-017-3505-0)
Supplement: Additional file 1: — Echocardiographic Protocol. The standardized echocardiographic protocol outlines all images to be obtained in order to meet the study objectives in the Cardiac Imaging Core. (DOCX 13 kb) [file 12885_2017_3505_MOESM1_ESM.docx]

**Additional file 1: Echocardiographic Protocol**

All echocardiographic imaging to be performed on the GE Vivid 7/E9. Observe the following settings:

- High frame rates necessary for colour TDI (>150 fps)
- 2D Frame rates should be 50-90 fps
- Record 4 beat loops
- Obtain BP (right arm) at the end of the study and enter into machine to calculate wall stress

Parasternal Long Axis View

- Zoom LVOT and aortic valve
- PLAX view with colour of aortic and mitral valves
- M-mode aortic valve for LVET/LAd and R-R interval
- VCFc
- PLAX RV inflow 2D and colour and CW Doppler
- RV outflow view from PLAX with colour and Doppler

Parasternal Short Axis View

- M-mode at level of mitral valve leaflet tips LV (SF and EF if possible)
- Colour Doppler PV and TV
- Obtain mean PA pressure when possible
- PW Doppler of main PA
- 2D PSAX views at MV/PAP/apical levels for 2D speckle strain
- Corresponding colour tissue Doppler PSAX at MV/PAP/apical level for strain (using appropriate TD Nyquist scale)

Apical Views (cross sectional areas and long axis dimensions/volumes)

- 2D 4 chamber view for bi-plane Simpson’s and 2D Strain
- 2D 2 chamber view for bi-plane Simpson’s and 2D Strain
- CALCULATE Simpson’s EF
- Colour MV/Aov and TV
- Obtain RVsp
- Obtain tricuspid valve inflow
- Obtain pulsed Tissue Doppler traces optimizing alignment in the basal lateral LV, the basal septal and basal lateral RV segment
- Obtain pulsed Doppler traces in the basal anterior and posterior segments on the 2-chamber view
- Obtain 4-ch apical view of LA/ RA: 2D+ color TDI
- Obtain 2-ch view of LA: 2D+ color TDI

Mitral valve Doppler/Pulmonary vein Doppler

- Record PW Doppler of Mitral inflow (MVe,a dt): between the valve leaflets (at tips of mitral leaflets)
- PW Doppler between inflow and outflow for IVRT and myocardial performance index
- Obtain Color-Doppler M-Mode of LV inflow with adequate baseline shift
- LV dp/dt: record CW Doppler of mitral regurgitation (RV dp/dt in single V)
- Record PW Doppler RUPV: optimize tracing

LVOT + AO valve Doppler

- Record PW LVOT Doppler
- Record CW Doppler through the aortic valve (gradient + aortic acceleration time)

Colour Tissue Doppler

- Broad sector views for colour TDI for LV dyssynchrony: include **4C + RV,** (RV free wall and septum, LV lateral wall and septum, **3C and 2C**-12 segments for analysis)
- Narrow sector views from 4-chamber for colour TDI of LV lateral wall, IVS and RV lateral wall for strain (narrow sector width= high frame rates ), from two chamber view obtain narrow sector of anterior and posterior wall

IVC/Hepatic veins

- Image and Doppler hepatic venous flow and abdominal aorta
- Image and Doppler of SVC from supra-sternal views - required in any patient who has or had a PICC)

At the end of study

- Obtain AFI
- Measure BP and measure WALL STRESS
